# School Gardens: A Qualitative Study on Implementation Practices

**DOI:** 10.3390/ijerph14121454

**Published:** 2017-11-25

**Authors:** Nele Huys, Katrien De Cocker, Marieke De Craemer, Marleen Roesbeke, Greet Cardon, Sara De Lepeleere

**Affiliations:** 1 Department of Movement and Sports Sciences, Ghent University, Watersportlaan 2, 9000 Gent, Belgium; Katrien.decocker@ugent.be (K.D.C.); Marieke.DeCraemer@ugent.be (M.D.C.); Greet.Cardon@UGent.be (G.C.); sara.delepeleere@ugent.be (S.D.L.); 2 Logo Gezond+, Baudelokaai 8, 9000 Gent, Belgium; marleen.roesbeke@gezondplus.be

**Keywords:** primary school, gardening, children, key members, vegetables

## Abstract

School gardens have beneficial effects on children’s dietary behaviors but information on its implementation is scarce. The current study aimed to gain insight in implementation practices of school gardens and in perceptions of key members and children towards a school garden. We conducted twelve interviews involving 14 key members and five focus groups with 38 children from fifth to sixth grade (10–13 years old) in four primary schools in Ghent (Flanders, Belgium). We analyzed the interviews and focus groups in NVivo, using thematic analysis. School gardens were mainly initiated to involve children in nature, not to improve vegetable consumption. Participants were positive about having a school garden, experienced facilitating factors (e.g., adaptability of the garden, having a person responsible for the garden), but also various barriers (e.g., difficulties with startup, maintenance during summer holidays and integration in the school curriculum) and suggested some solutions (e.g., involving external organizations and parents, expanding the garden) and motivating factors for children (e.g., colorful plants, use of gloves). In order to improve implementation and to contribute to children’s health, future school gardening projects should take the recommendations of key members and children into account.

## 1. Introduction

Schools are convenient settings to implement health promoting interventions targeting children [1,2] because of the continuous, intensive contact with children [3] and the opportunity to reach almost all children [4]. One approach that can contribute to health behaviors of children is the implementation of school gardens, defined by the Food and Agricultural Organization of the United Nations as “cultivated areas around or near to primary schools, which can be used mainly for learning purposes but could also generate some food and income for the school” [5]. Gardening projects in schools give primary schoolchildren the opportunity to grow and harvest their own fruit and vegetables [6,7]. The systematic review of Ohly et al. (2016), which aimed to examine the health and well-being impacts of school gardening, shows that school garden interventions have beneficial effects on children’s willingness to taste vegetables, on preferences for fruits and vegetables and on knowledge of and attitudes towards food [8]. Furthermore, some studies showed a positive effect on fruit and vegetable consumption [9,10]. However, these health impacts can only be reached by a successful implementation and when key members and children are motivated to engage in the school garden activities. The review of Ohly et al. also identified some factors that influence the success and sustainability of the school garden. The most important success factors were the support from stakeholders (staff, gardening specialists, volunteers and local organizations), the integration within the curriculum, the supportive and inclusive environment (i.e., all children can participate), connection with cultural heritage and local foods, a garden committee, and links with the wider community. The recruitment of volunteers, increased pressure on workload and financial challenges, were identified as difficulties [8]. Findings of the study of Ohly et al. [8] were only derived from studies in the UK, the USA and Australia, so evidence from other European countries is lacking, which limits the generalizability of school gardening practices. In Flanders (Belgium), the population density is higher than in the USA and Australia (481.43 inhabitants/km^2^ compared to respectively 33.2 inhabitants/km^2^ and 3 inhabitants/km^2^) (data.worldbank.org/indicator/EN.POP.DNST), which results in smaller open spaces at schools. Therefore, school gardens in Flanders (Belgium) will be implemented on smaller surfaces, which could entail different practices, problems and barriers. To our knowledge, no studies about school gardening were conducted in Belgium, although some primary schools in Flanders (Belgium) hold a school garden. School gardens in Flanders are not part of a larger gardening project where information and guidance on the maintenance and use of the garden is provided, which could also result in different perceptions on the implementation compared to the qualitative results in the review of Ohly et al. (2016) [8].

Therefore, the present study aimed to gain insight in implementation practices of school gardens, including facilitating factors and barriers and to gain insight in the perceptions of key members and children towards school gardening in a Belgian context. Insight in these factors may inform future school gardening projects.

## 2. Materials and Methods

### 2.1. Participants

In November 2014, we contacted all primary schools with a school garden in the region of Ghent (Belgium) (*n* = 34) by email or phone to participate. A total of five schools agreed to participate (response rate (RR): 14.7%). Main reasons to decline were lack of time and having too many requests to participate in studies. We excluded one school because of its special education program (i.e., education for children who are unable to attend regular education for several reasons) which could cause that the school garden differs too much from that in regular schools. Of the four included schools, two schools were located in the city center and two schools were located in the suburban region of Ghent. In each school, we appointed a contact person who was interested in the study. He/She recruited key members who were involved in the school garden (teachers, a parent, a headmaster and two ‘environmental care at school’-teachers) and children from the fifth and sixth grade (10 to 13 years old) who already had experience in school gardening and who were interested in a discussion on this topic. In December 2014, we distributed written consent forms to the selected key members and children. Key members who gave written consent and children whose parents gave written consent, were included in the study. A total of 14 key members (RR: 38%; 79% women) participated in the interviews. Furthermore, 38 children (RR: 11%; 47% girls) participated in the focus groups. In each school, three to four key members participated in the interviews and six to sixteen children participated in the focus groups. Detailed information on the participants is described in Table 1.

### 2.2. Materials

In this qualitative study, we conducted interviews with key members and semi-structured focus groups with children. In key members, conducting interviews was selected over focus groups since their time schedules did not allow to gather enough key members to organize focus groups. An interview guide and a focus group guide (Table 2) were developed consistent with recommended interview and focus group methodology [11]. The guides were formulated to investigate the implementation practices of the school garden, and key members’ and children’s perceptions on school gardening. More detailed optional questions were asked to stimulate the discussion or to gain more insights in the theme.

### 2.3. Procedure

In total, we conducted ten individual interviews with key members, two interviews with two key members and five focus groups with each six to nine children at school until saturation was reached. We held all interviews and focus groups in Dutch and interviews and focus groups lasted respectively 10 to 20 min and 30 to 45 min. One interviewer conducted interviews and a moderator and co-moderator led the focus groups.

### 2.4. Ethical Statement

This study was conducted according to the guidelines laid down in the Declaration of Helsinki and all procedures involving human subjects/patients were approved by the Ethics Committee of the University Hospital of Ghent (project 2014/1294). Written informed consent was obtained from all participating key members and parents of the participating children.

### 2.5. Data Analysis

We audiotaped focus groups and interviews and made verbatim written transcriptions of the focus groups and interviews and entered them into the qualitative research software NVivo 10 (QSR international, Melbourne, Australia) to analyze the data thematically in several phases. First, codes were developed based on the focus group and interview guides. Secondly, additional codes were developed during the transcription of the audio recordings according to the responses and the themes which arose frequently and were relevant to the aim of the study. Coding doubts or disagreements were discussed until full consensus was reached.

## 3. Results

Quotes of key members and children can be found in Table 3.

### 3.1. Implementation Practices

#### 3.1.1. Goals

Key members reported that the idea to start a school garden mainly came from teachers and the school working group ‘Environmental Care at School’. The following goals for the establishment of the school garden were mentioned: teaching the children about (forgotten) vegetables, teaching them the growth process and origin of the plants and giving children the experience of working in a garden. It was indicated that learning about gardening and vegetables may particularly be important for children who live in the city because they do not experience gardening at home. During the interviews, some key members suggested to add ‘improving children’s health’ to the goals of the school garden. They believed that a school garden can contribute to the health of children by teaching children that they can grow their own healthy food and by influencing their consumption behavior.

#### 3.1.2. Characteristics of the Garden

Two schools had their school garden only since one year, whereas the two other schools had the garden already for seven to ten years. The school garden was adapted to the space and needs of the schools. Two schools wanted to start small and therefore had a garden in planters. The surface of these gardens was 4 to 6 m^2^. The other two schools had a garden in solid ground with a surface of 50 to 160 m^2^.

#### 3.1.3. Organization of the Garden

All four schools had a working group ‘Environmental Care at School’, which is an initiative of the Flemish Department of Environment, Nature and Energy. This initiative supports primary and secondary schools to make the school an eco-friendly and sustainable learning environment. Some schools bought seeds and materials for their school garden with funding from this initiative. Additionally, schools received materials for the school gardens from parents or other gardening projects such as “Watch them grow” (a project that aims to encourage people to create a green, healthy and pleasant environment), Circa Gent (the cultural center of Ghent that develops several cultural projects) and Velt (an association that provides information about ecological gardening). Key members mentioned that support from such organizations or community projects was useful for the startup of a school garden. Furthermore, in one school, the help of a parent was important at startup. Key members mentioned they found it important to have information about the care of the plants which could be found in videos, books, calendars…

Working in the school gardens was organized in voluntary moments at lunch break or afternoon break and class bound moments with a fixed schedule and a rotation system per grade or class when teachers worked in the school garden with the children. In different schools, teachers reported that they tried to integrate the fruit and vegetables theme in their classes e.g., by making soup, following the growth process of a plant, learning about seasonal fruit and vegetables and ecological footprint. However, teachers mentioned they spend little time on these subjects, because they do not often occur in the curricula.

The harvest of the school garden was used differently in all schools. Some key members reported they used the harvest systematically in class, while others exposed the vegetables in the staff room or did not use the harvest because the largest part was ripe in summer. Key members suggested some possibilities to better use the harvest, such as making jam or dressings, having tastings. Additionally, three schools sometimes sold parts of the harvest to parents. The income generated from the sale was used for gardening materials for the next school year.

#### 3.1.4. Staffing

All schools had a person responsible for the garden and key members indicated this was essential for its maintenance. This person could stimulate and accompany other key members and could ensure that all the work is done in time. Generally, teachers who were part of the working group “Environmental Care at School” took responsibility and mentioned that it could be motivating if they would receive exempt hours for this task.

### 3.2. Perceptions on School Gardening

#### 3.2.1. Perceived Effects

Key members perceived some effects on knowledge about vegetables among children who work with the school garden. They mentioned that children memorize a lot of information while gardening and that working in a school garden is a potential means to have a first acquaintance with vegetables. Children themselves mentioned they get to know new vegetables, become aware of the origin of vegetables, learn how different plants look like, what they need to grow, how to garden and how the different vegetables taste.

Furthermore, key members perceived some effects on the attitudes of children towards vegetables: more children taste the vegetables when they have grown them themselves. Possible explanations given were a lower threshold to taste by following the growth process, vegetables being more present in children’s everyday environment and knowing where the vegetables are originated. Children confirmed these findings by mentioning that they had increasing interest in vegetables by being involved in the school garden.

Lastly, key members expected no change in vegetable consumption of children. The main reason was that the parents were mostly not involved, while according to the key members the home environment is most important in the development of consumption patterns. Only one school tried to involve parents by giving children little plants to take care of at home together with their parents. Other reasons for the lack of change were: children did not know the importance of eating vegetables, did not use the school garden long enough, did not spend enough time in the school garden and did not consume vegetables because of the low harvest. Also, the majority of children reported no changes in their vegetable consumption.

#### 3.2.2. General Perceptions

Key members and children were generally positive about the school garden. They also indicated that parents were positive about the garden at school.

Key members would recommend a school garden project to other schools because it has an added value for children and is a feasible project for schools. On the other hand, key members encountered some difficulties at startup and the school garden can be seen as a burden for teachers. For the latter, a solution was proposed: involving parents for maintenance and supervision of the school garden.

Furthermore, children and key members suggested that children should work more often in the school garden to keep the work interesting and to make it possible to use the harvest to make dishes they can eat themselves. Regardless of the size of their current gardens, both children and key members indicated that a larger school garden could give the opportunity to involve more children. However, there is often no space at school to expand the garden.

Also, key members indicated that it was difficult to maintain and/or use the garden during winter and school holidays. In one school, key members reported that planting winter vegetables enabled them to continue using the garden during winter. As a lot of vegetables are ripe during the summer holidays (July and August), key members stated they tried to ensure that most of the vegetables could be harvested before holidays or that the school garden was maintained by parents and teachers during holidays.

Children indicated that they liked the fact that the school garden brought greenery and colors at school, but it was mentioned they wanted the setting to be even more colorful. They suggested to plant more colorful vegetables or flowers or to paint the garden planters. Additionally, children were excited about cooking with the homegrown vegetables.

Most children were enthusiastic about being outside while working in the school garden and thought that their parents were positive about them being outside. Other children did not like to be outside, especially in certain weather conditions (rain, very cold, very sunny) or right after play time.

Most children also reported that it was fun to work with their hands. Getting dirty hands was part of working in the school garden and they did not need gloves for it. The latter was mentioned as a solution for children who reported they did not like to make their hands dirty.

Although in general, children were positive about the school garden, they did not mention the school garden very often at home or to friends because they did not work enough in the garden and found it an awkward theme to discuss with friends.

Children also mentioned some problems regarding the school garden: other children sometimes walk over the plants, destroy plants or pull them out, whereby plants never sprout. Suggested solutions regarding these problems were placing a fence around the garden, placing a greenery for vegetables that need more care, giving a punishment when children go into the school garden when it is not allowed and better and more supervision from teachers.

## 4. Discussion

The findings of this qualitative study on school gardens in Flanders (Belgium) provide insights in the implementation practices of school gardens and into the perceptions of key members and children towards a school garden. More specifically, the study gives insight into the practical organization of a school garden in schools that have little open space to implement a school garden, compared to schools in, for example, countries such as the USA and Australia [8]. This additional geographical diversity in school garden research leads to more specific recommendations for schools in this situation, beyond the recommendations in the review of Ohly et al. (2016) [8]. In general, key members and children were positive about the garden at school, but some findings should be given special attention.

A first finding is that schools in the present study started the school gardens to involve children in nature and not to encourage children to eat more fruit and vegetables. Studies in other countries also showed that existing school gardens are primarily used for environmental education and not to have an impact on children’s health [12,13]. However, the review of Ohly et al. (2016) states that school gardens can have positive impacts on children’s health and wellbeing [8], not only on fruit and vegetable consumption, but also on physical activity. Promising effects of school gardening on physical activity were also reported in the studies of Hermann et al. (2006) and Wells et al. (2014) [14,15]. Since the Framework for Successful Implementation of Durlak and DuPre [16] states that findings from research should inform the implementation, it could be of added value if schools also introduced a healthy lifestyle, focusing on both nutrition and physical activity, when starting a school garden. Key members in the present study agreed that encouraging children to eat more vegetables could be an additional aim of the school garden, although they did not perceive positive effects on fruit and vegetable consumption as parents were not involved and children did not spend enough time in the school garden. However, they did perceive positive effects on children’s knowledge and attitudes towards vegetables, which can be important for the success of the school garden. Also, the Framework for Successful Implementation states that stakeholders need to believe that a project will have beneficial effects to succeed [16].

Secondly, school gardens in the present study were mainly funded by the Flemish government. However, this funding was supplemented with gifts from parents, other projects and profits from sales of the harvest. Amplifying financial resources with other initiatives (e.g., fundraising events, donations from local organizations [8]) could be important, as funding is one of the success factors of implementation [16].

Another finding is that key members in the present study appreciated the help of external organizations (e.g., Velt, circa Gent, “Watch them grow”) at start-up, which was also mentioned in the study of Hazzard et al. (2011). These organizations can provide technical assistance and contribute to key member’s knowledge and experience [17], which is often lacking [13,18]. The intervention’s coordination with other agencies is also a factor that affects the implementation process [16].

Furthermore, key members suggested to involve parents or other volunteers to work with the children in the garden to diminish the experienced burden for teachers [13,17,19]. Moreover, parents, children and local organizations can help maintain the garden during holidays, which was mentioned as a problem in the schools. Involving parents will also be necessary to increase the effects on fruit and vegetable consumption, as literature shows that parents play a major role in the vegetable consumption of children [20,21,22]. Parents can be invited to visit the garden during a parents’ evening, can be sent newsletters about the health benefits of eating fruit and vegetables and possibilities to increase intake at home or can do homework tasks together with their children (e.g., writing down their favorite vegetable dish) [23,24]. Key members and children suggested that a larger school garden would be beneficial, but there was a lack of space to expand the garden. Therefore, the involvement of communities can also help schools to overcome this problem as communities can provide space such as parks or open spaces to expand the garden [25].

The school gardens are adaptable to the available space of schools, as they can exist in solid ground or in planters. This is also in line with the Framework of Successful Implementation that states that implementation can be stronger if a program is adaptable to the needs of providers [16]. An extra garden in planters (which is as effective as a garden in solid ground [26]) can be a solution when schools lack space to involve all children, which was a major problem in the present study as schools were located in a (sub)urban region. The study of Oxenham and King, which experienced the same difficulties, advised schools with limited space to also use community places to garden, e.g., in a park or open space nearby the school [25].

Another finding is that key members in the present study indicated difficulties to integrate the work in the school garden in the curriculum, which is in line with previous research [13,18,27]. However, the framework of Durlak and DuPre states that incorporating a new program in the existing practices is important to build organizational capacity [16]. Suggestions to integrate the school garden in core curriculum subjects were given in previous projects: growth measurement of the plants in mathematics, finding out the cost of the garden, creating a fictitious business, relating songs and stories to the school garden… [19,28]. Furthermore, a school garden can be linked to the Flemish educational attainment goals or developmental objectives, defined by the Minister of Education, about science and technology, as nature, health and environment are main topics in this area (e.g., pupils can independently perform basic operations when caring for animals and plants from their environment) [29]. The integration of school gardens in the curriculum will be important to enhance the involvement of children in the garden, which is important to keep them motivated [27]. Teachers may be more encouraged when the school garden will no longer be an ‘extra’ task in their job.

A last finding is that key members found it important to have a person responsible for the garden. Also, the Framework for Successful Implementation suggests the allocation of a ‘program champion’ (i.e., a person who can inform and support key members [13,17]) to build organizational capacity [16]. A suggestion of the key members in the current study is that Environmental Care at School-teachers are granted exempt hours so they can become responsible people who work with the children in the school garden.

### 4.1. Implications for Practice

This study has several implications for school garden practice. First, it seems important to focus on the involvement of children in nature, as teachers think this is important. However, the potential to work on a healthy lifestyle (e.g., healthy food and physical activity) while working in the school garden needs more emphasis. Nevertheless, due to limited space in schools in Flanders, it is only possible to predominantly focus on healthy food instead of physical activity. The lack of focus on a healthy lifestyle seems a missed opportunity, while this could increase the motivation of the different stakeholders and lead to more support and funding for garden projects from other sectors, such as the health sector. Second, solutions have to be found to minimize the organizational burden of a school garden. A possible strategy is to provide sufficient external support at start-up. Third, to increase effects on health behaviors of children, it will be important to involve parents and to make the school garden attractive to children, by making the garden colorful, cooking with the harvest and preventing access to the garden from children who destroy the plants. Furthermore, it is recommended to better integrate working in the school garden in the core curriculum and to search for more space for the school garden in order to increase the harvest and to give more children the opportunity to work in the garden. Taking into account all of these factors will motivate both key members and children, but also other stakeholders and may eventually lead to better health in children.

### 4.2. Limitations

This study gives valuable insight in the implementation practices and perceptions of key members and children towards a school garden, but also has some limitations. First, the low school response rate (14.7%) may have resulted in selection bias. Since time constraint was the main reason not to participate, it is possible that teachers in non-participating schools have different perceptions on the burden of a school garden. However, differences in school characteristics such as school system and size, number of pupils and number of teachers were limited. Consequently, it can be assumed that differences in perceptions between participating and non-participating schools are limited. Second, all included schools are located in a (sub)urban region (Ghent), which may limit generalizability to schools in rural regions. A third limitation is that participating children were selected by the school contact person, which could have resulted in bias as it is possible that only the most motivated children or children with the most experience in the school garden were included. Fourth, in focus groups the most dominant people get most of the talking which may give a distorted picture of the opinion of the group. However, in this study, the moderator and co-moderator tried to involve all children in the discussion. Finally, children also influence each other’s answers so that individual opinions may have been different.

## 5. Conclusions

Although the schools in the present study have different school gardens in terms of type (garden in solid ground or garden in planters) and surface (4 to 160 m^2^), the perceptions towards a school garden and perceived problems and barriers for the implementation of a school garden were overall similar. In general, key members and children were positive about a school garden, but encountered some practical issues that needed to be solved to improve efficiency. The findings of the study have led to recommendations and tips for future school garden practices.

## Figures and Tables

**Table 1:** Characteristics of participating schools.

	School 1	School 2	School 3	School 4
Location of school in Ghent	City center	Suburban region	Suburban region	City center
Number of children in 5th to 6th grade	16	75	61	194
Number of teachers in 5th to 6th grade	6	5	6	20
Participating children	2 focus groups: one with 7 children and one with 9 children	1 focus group with 6 children	1 focus group with 8 children	1 focus group with 8 children
Participating key members	2 teachers and 1 responsible person (parent)	4 teachers	2 teachers and 1 responsible person	3 teachers and the headmaster

**Table 2:** Focus group guide for children and interview guide for key members.

Theme	Questions for Children	Questions for Key Members
Contextual information on the school garden and perceptions on implementation	What does the school garden look like? How long have you been working in the school garden? What fruits or vegetables do you grow in the school garden? Do you want to grow other fruits or vegetables in the garden? Do you work with the teacher in the garden or do you work alone or with friends? Would it be good to work alone in the garden? Why? Would it be good to work with friends in the garden? Why? Would it be good to work with the teacher in the garden? Why?	How did the idea grow to start a school garden? How long is the school garden already in use? Is the school garden part of a broader health policy at school? Are there other actions at school to work on children’s health? Why do you choose to work with a school garden? Who is working with the school garden? Teachers: all teachers? Children: what age, which classes? All children in class or only a few children? Are parents involved? Why, why not? Can you, as a teacher, choose to work in the school garden or is it an obligation? When do teachers and children work in the school garden? During the complete school year? Also in holidays or in winter? During classes? Are there specific lessons about the garden? During breaks? During leisure time? Who provides the seeds or plants for the garden? Which vegetables or fruits do you grow in the garden? Do they all succeed? Why, why not? What happens with the harvest? Processed in class, at school? Something else?
Perceived effects of a school garden	5. Do you like fruit and vegetables more now? 6. Do you eat more fruit and vegetables now? And why? 7. Have you learned more about fruit and vegetables by working in the school garden? What have you learned?	9. Do you perceive benefits for children who work in the school garden? Which ones?
Attitudes towards the school garden and the development of school garden projects	8. What do you think about working in the school garden? What do you like about it? What don’t you like about it? 9. Do you think it is good to be outside when working in the garden? Why? 10. Do you mind that your hands get dirty when you work in the garden? Why? What bothers you about it? 11. Do you talk about the school garden with your friends or at home? What do you talk about then?	10. How do you experience working with the school garden? 11. The purpose of a school garden project can be increasing fruit and vegetable consumption. What do you think about this goal? 12. Do you perceive barriers of a school garden? Which ones?
Ending	12. Is there anything else you want to say about the school garden?	13. Is there anything we should have talked about but did not?

**Table 3:** Quotes of children and key members.

Theme	Quote
**Implementation Practices**	
Goals	Teacher: “In the school garden we try to teach the children the growth process of vegetables, taking care of plants, and we also focus on the origin of plants, as children often lack knowledge on this”. Teacher: “We do not really focus on this goal (encouraging children to eat vegetables), but I think we indeed have to focus more on the fact that you can eat your own vegetables and you can even cook with them”.
Perceived effects	Teacher: “My own children go to this school and my oldest son tells me that he is interested in gardening and I must admit that everything the teachers tells lingers”. Child: “We learn what plants need to survive, how they grow and that they are all different”. Teacher: “If they (the children) grew it (vegetables) themselves and they saw it growing, they are motivated to taste and eat it”. Teacher: “Working with the school garden will not make a lot of difference as we don’t involve the parents. It is necessary that at home there’s a positive attitude towards vegetables”. Child: “I do eat more vegetables. I trust the fresh vegetables more than vegetables that came a long way to the shop”.
Characteristics of the garden	Teacher: “We subscribed to the contest of “Watch them grow” and we got a starters pack: a planter of 1 m^2^ and lots of information and movies”.
Organization of the garden	Teacher: “Starting a school garden was an idea of a colleague. We were looking for something to do within our Environmental Care at School-project, to plant and sow with children”. Teacher: “I’ve got information from videos, but to have a good growth process of the plants, it is necessary to have some experience in gardening or that you search lots of information”. Teacher: “The maintenance of the school garden and sowing some more plants was done at lunch break, once a week, with a parent”. Teacher: “Sometimes it occurs in a theme of the subject ‘world orientation’, but if you (as teacher) work very method bound, it is very difficult to integrate the fruit and vegetables theme”. Teacher: “We ensure that we adapt our crops so that few are ripe in summer holidays and sometimes there are people coming to weed or to water the plants when it is very dry”. Responsible person: “I e-mail teachers to announce what is ready to use and then they see if they can fit it in their schedule, and they compromise on who harvests which plants and who uses them”.
Staffing considerations	Teacher: “It is important that there is a person who stimulates the whole process, who says: “Now it is time to do this and ‘that’ person is going to do it…””.
**Perceptions on School Gardening**	
Positive attitudes of key members	Teacher: “It is fun and I think it is feasible. This is perfect and we actually had a good harvest from those planters”.
Barriers of key members	Teacher: “A lot of teachers are not keen to do it (=working together with the children in the garden), because it is seen as a burden and because they need to maintain it over the year”. Teacher: “We have a relatively small area within the school with a lot of children, so it’s not easy to make space for more greenery”.
Positive attitudes of children	Child: “I think it was nice to do something new and something different during class than what we otherwise do”. Child: “I don’t think we need gloves for those few things because it is nice to have your bare hands… Yeah, get dirty”.
Barriers of children	Child: “If there is not a lot of work, I don’t really like it”. Child: “When it’s cold or it rains, I like to be inside instead of being outside in the cold”. Child: “Most of the time, the plants are already broken-down because of the toddlers who pull them out, yes, they damage it”.
